# Mitochondrial retrograde signaling inhibits the survival during prolong S/G2 arrest in *Saccharomyces cerevisiae*

**DOI:** 10.18632/oncotarget.6406

**Published:** 2015-11-27

**Authors:** Anna N. Zyrina, Maksim I. Sorokin, Sviatoslav S. Sokolov, Dmitry A. Knorre, Fedor F. Severin

**Affiliations:** ^1^ Faculty of Bioengineering and Bioinformatics, Moscow State University, Moscow, Russia; ^2^ Belozersky Institute of Physico-Chemical Biology, Moscow State University, Moscow, Russia

**Keywords:** cell cycle arrest, telomere dysfunction, retrograde signaling, Rtg pathway, mitochondria

## Abstract

Cell senescence is dependent on the arrest in cell cycle. Here we studied the role of mitochondrial retrograde response signaling in yeast cell survival under a prolonged arrest. We have found that, unlike G1, long-term arrest in mitosis or S phase results in a loss of colony-forming abilities. Consistent with previous observations, loss of mitochondrial DNA significantly increased the survival of arrested cells. We found that this was because the loss increases the duration of G1 phase. Unexpectedly, retrograde signaling, which is typically triggered by a variety of mitochondrial dysfunctions, was found to be a negative regulator of the survival after the release from S-phase arrest induced by the telomere replication defect. Deletion of retrograde response genes decreased the arrest-induced death in such cells, whereas deletion of negative regulator of retrograde signaling *MKS1* had the opposite effect. We provide evidence that these effects are due to alleviation of the strength of the S-phase arrest.

## INTRODUCTION

Mechanisms of mutual coordination between nuclear DNA replication and mitochondria functioning are still an opened question. Several studies showed that replication of mitochondrial DNA (mtDNA) occurs at all stages of cell cycle [1, 2] and arresting cells in cell cycle does not prevent mtDNA replication [3, 4]. Conversely, there are evidences of reciprocal regulation between mitochondria and cell cycle controlling machinery. It was shown that the main cyclin-dependent kinase (yeast Cdk1) controls the assembly of TOM complex to accommodate for the increased energy demand during S-phase [5]. Moreover, the structure of mitochondrial network in some cell types is changing to reflect the requirements of a specific cell cycle stage [6]. Several works pointed at a possible existence of the mitochondria-dependent checkpoints [7–9].

At the same time, mitochondria play a crucial role in cell senescence (see for review ref. [10]). Cell senescence emerges due to growth stimulation of cells unable of cell cycle progression (see for review refs. [11, 12]). The activity of mTOR pathway is required for such geroconversion (see for review ref. [13]). One of the causes of cell cycle progression inhibition can be telomere dysfunction [14, 15]. Yeast *Saccharomyces cerevisiae* cells with non-replicated telomeres arrest in S/G2 phase. The arrest is accompanied by ROS formation and activation of a protease with caspase-like activity [16]. Long term arrest irreversibly blocks further proliferation, while inhibition of mTOR pathway prevents the viability loss [17]. This shows a striking similarity of arrest-induced irreversible proliferation block (death) in yeast and senescence in higher eukaryotic cells proposed earlier [18]. Yeast cells lacking mitochondrial DNA are also resistant to S/G2 arrest-induced death [16]. Apparently, as the loss of mitochondrial DNA causes major changes in the physiology of yeast cells, there could be multiple reasons for the increased viability during prolonged arrest in S-phase. Qi et al. [16, 17] suggested that the increased viability was due to suppression of mitochondrial ROS production caused by the loss of functional respiratory chain. Consistent with this, it was shown that antioxidants N-acetylcysteine and ascorbic acid prevented yeast cell death induced by telomere dysfunctioning [17]. The experiments were performed with extremely high concentrations of the antioxidants (0.1–1 M). The authors favored the possibility that the antioxidants suppressed the elevated ROS production. However, another possibility is that this was because low levels of ROS are necessary for normal progression of the cell cycle [19]. For this reason, high antioxidants concentrations may prevent death by arresting cells in G0/G1 phase. At the same time, the mitochondria dysfunction (i.e. loss of mtDNA) was shown to induce activation of specific mitochondria-to-nucleus (Rtg) signaling pathway. Rtg pathway includes Rtg2p sensor for mitochondria dysfunction [20, 21] and two transcription factors Rtg1p and Rtg3p which are negatively regulated by multiple kinase suppressor Mks1p [22]. This pathway is activated in cases of mitochondrial dysfunctions, e.g. loss of mitochondrial DNA, it induces the expression of the genes responsible for the bypass of metabolic reactions which require functional mitochondrial respiratory chain (see ref. [23]). The activation of Rtg pathway leads to a hormetic induction of stress-response genes [24] and thus could provide an additional protection for S-phase arrested cells. Indeed, it was recently shown that the deletion of Rtg genes decrease the survival of replicatively old yeast cells under stress conditions [25]. It was proposed that Rtg1p, Rtg2p and Rtg3p are the most likely candidates for a specific mitochondrial checkpoint, which prevents cell cycle progression when mitochondria are severely damaged [7].

Here we compare the roles of mitochondria in yeast cells committed to long arrest at different stages of the cell cycle. To discriminate between the direct and the signaling consequences of *Rho0* transformation in the arrest-induced death we focused on a possible role of Rtg pathway in this process.

We provide evidence that, similar to S-phase, the prolong delays at metaphase or anaphase but not in G0/G1 also cause cell death. We found that the loss of mitochondrial DNA increases the survival of yeast cells by increasing the duration of G1 phase regardless of Rtg-pathway activation, whereas Rtg signaling affects the survival in the S-phase. We found that inactivation of Rtg signaling alleviates the strength of S-phase arrest induced by telomere dysfunction and thus increases the survival.

## RESULTS

**Figure 1:**
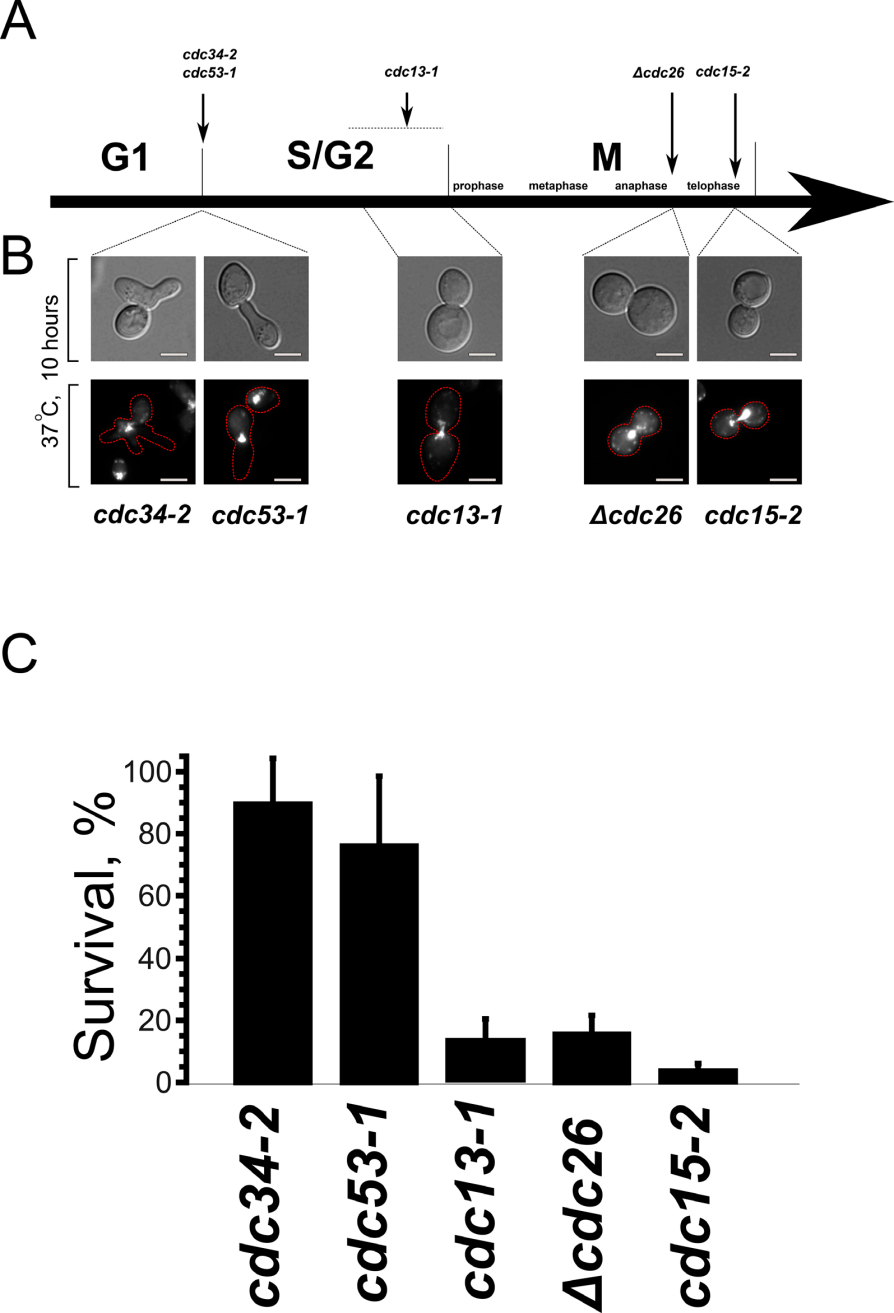
Prolong arrest in interphase, unlike the division phases, does not cause cell death (**A**) Schematic representation of the arrest points of the temperature-sensitive mutants used in this study. (**B**) Phenotypes of the mutant cells after a prolong arrest at the non-permissive temperature. Upper panels, DIC; lower panes, DNA staining (DAPI). (**C**) Survival of the indicated mutants after 10 hour arrest presented as colony forming units. Data are represented as mean +/− SEM.

First, we asked whether the cell cycle delays caused by the mutations other than the one preventing telomere replication can also kill the cells. To do this we used the strains carrying temperature-sensitive mutations in *CDC13*, *CDC34*, *CDC53*, *CDC26* or *CDC15*. *cdc13-1* strain carries a mutation in telomere binding protein, the same mutant was used by Qi and co-authors [16, 17], we used the strain as a positive control (Figure 1A, 1B). Cdc34p and Cdc53p are subunits of SCF ubiquitin-protein ligase complex, at non-permissive temperature the mutants arrest in G1 (Figure 1A, 1B and [26, 27]). Cdc26p is a non-essential subunit of Anaphase Promoting Complex, at non-permissive temperature the null mutant arrests at metaphase (Figure 1A, 1B, and [28]). Cdc15p is a protein kinase, a constituent of mitotic exit network, upon the temperature shift the mutant arrests with elongated mitotic spindles and undivided nuclei (Figure 1A, 1B and [29]).

Figure 1C shows the survival of the temperature-sensitive mutants after a prolong delay at non-permissive temperature. It appeared that, despite the significant changes of cell morphology, the mutant cells which were delayed in G1 retained their ability to form colonies after the shift to the permissive temperature. On the contrary, the viability of *cdc13-1*, *Δcdc26* and *cdc15-2* mutants was suppressed by the delay.

**Figure 2:**
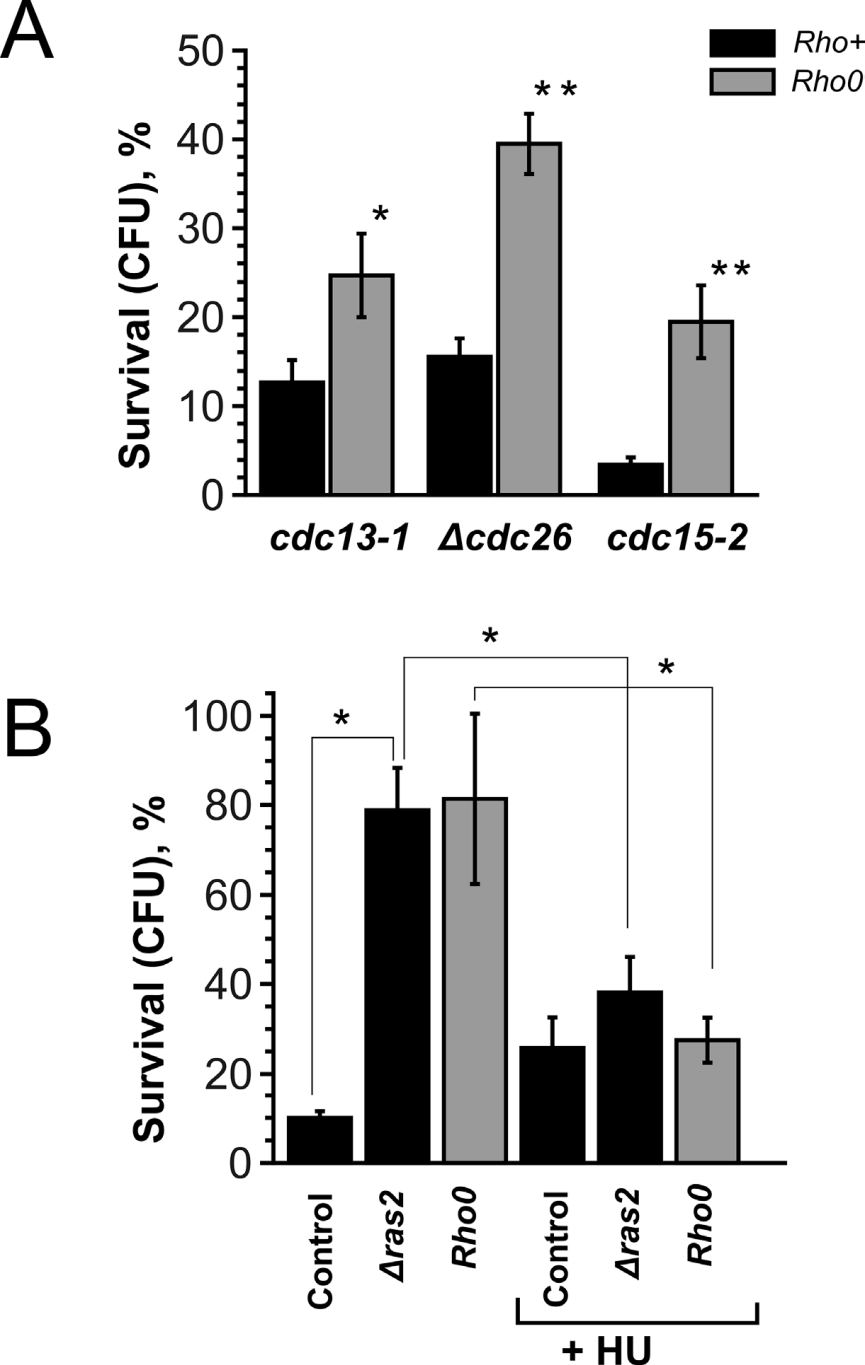
*Rho0* transformation and *RAS2* deletion improve the survival by increasing the duration of G1-phase (**A**) Survival rates of various temperature-sensitive mutants and their *Rho0* versions after 10 hour arrest. (**B**) Survival rates of *cdc15-2* (control), *cdc15-2 Δras2* and *cdc15-2 Rho0* cells with or without pre-synchronization in the S-phase. Data are represented as mean +/− SEM.

Next we addressed possible similarities between the death processes in *cdc13-1*, Δ*cdc26* and *cdc15-2* cells. The previous studies on *cdc13-1* [16, 17] pointed at mitochondrial involvement. Thus, we generated *Rho0* versions of *cdc13-1*, Δ*cdc26* and *cdc15-2* mutants and compared the survivals with the original strains. It appeared that, similar to *cdc13-1*, the loss of mitochondrial DNA protected Δ*cdc26* and *cdc15-2* mutants from the delay-induced death (Figure 2A).

**Figure 3:**
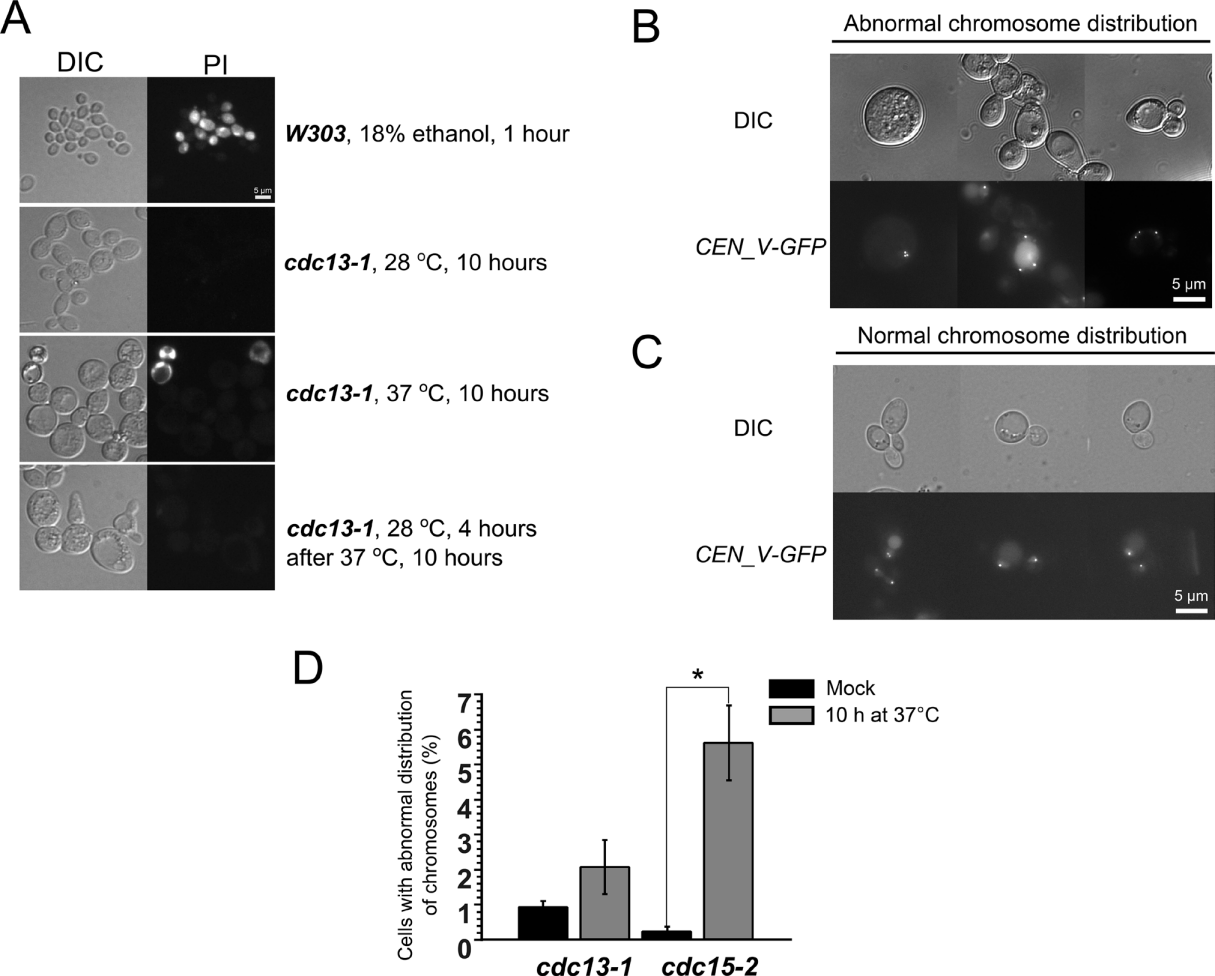
Prolong (10 h) incubation of *cdc15-2* mutant at non-permissive temperature causes chromosome V missegregation (**A**) Representative images of yeast cells subjected to stress and stained with propidium iodide. (**B** and **C**) Examples of *cdc15-2* cells with normal and abnormal distribution of CEN_V-GFP signal, respectively. The cells were analyzed after 24 h recovery at permissive temperature. (**D**) Quantification of the missegregation events in *cdc13-1* and *cdc15-2* mutant cells. Data are represented as mean +/− SEM.

To look into the mechanism of the delay-induced cell death we screened for genes deletions of which increase the survival (see Materials and Methods section for details). It appeared that *cdc15-2 Δras2* double mutants displayed the survival rates similar to the ones of *cdc15-2 Rho0* cells (Figure 2B). Ras2p is a small GTPase, it regulates adenylate cyclase activity and transcriptional responses to nutrient limitation [30]. We stained the cells after the incubation at non-permissive temperature with a fluorescent dye propidium iodide (PI), which accumulates only in dead cells. Interestingly, the bulk of *cdc13-1*, Δ*cdc26* and *cdc15-2* cells were PI-negative even after 10 hours of arrest followed by 1 hour of recovery (Figure 3A). We reasoned that the failure to form colonies could be explained by major chromosomal rearrangements which are incompatible with further proliferation. To test this hypothesis we took advantage of a construct which marks the region proximal to the centromere of chromosome V with fifty copies of GFP [31]. CEN_V-GFP construct was introduced *cdc15-2* mutants, the cells were grown and then shifted to the non-permissive temperature as in materials and methods, then shifted to permissive temperature for 24 hours and then the centromeres were visualized under the microscope (Figure 3B, 3C). The percentages of cells with abnormal numbers of chromosome V (three GFP dots or more) were calculated. It appeared that ten hour incubation at the non-permissive temperature significantly increased chromosome missegregation in *cdc15-2* (Figure 3D).

Thus, cell death in *cdc15-2* mutant is likely to be caused by major chromosomal rearrangements. At the same time, deletion of *RAS2* is known to tighten G1 arrest of the cells under unfavorable conditions [30]. *Rho0* transformation was also shown to have a similar effect, i.e. to delay G1- to S-phase transition [32]. As, according to our data (see above), a delay in G1 phase does not cause yeast cell death, we reasoned that the effects of *RAS2* deletion could be explained by G1 elongation. To test this, we synchronized the cells in the S-phase by incubating them in hydroxyurea before shifting them to the non-permissive temperature. It appeared that such synchronization removed the protective effects of both *RAS2* deletion and *Rho0* transformation in *cdc15-2* mutants (Figure 2B).

**Figure 4:**
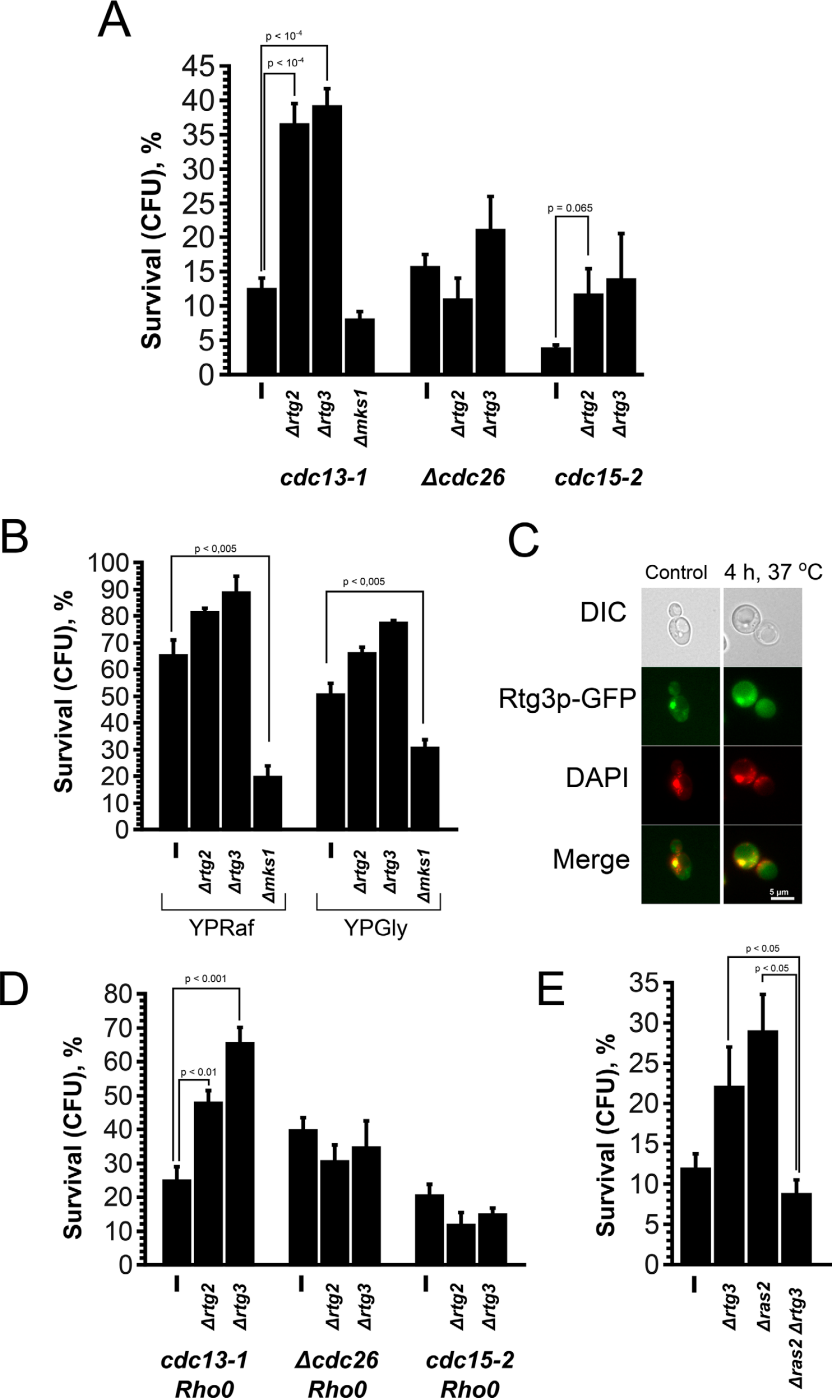
Deletion of Rtg genes and *MKS1* have opposite effects on the survival of *cdc13-1* cells (**A**) The survival rates of *cdc13-1*, Δ*cdc26* and *cdc15-2* single mutants and their Δ*rtg2*, Δ*rtg3* and Δ*mks1* versions after 10 h arrest at non-permissive temperature. (**B**) The survival rates of *cdc13-1*, *cdc13-1 Δrtg2*, *cdc13-1 Δrtg3* and *cdc13-1 Δmks1* mutants grown either on YP-raffinose or on YP-glycerol media. (**C**) Images illustrating co-localization of Rtg3-GFP signal with nuclear DNA in *cdc13-1* cells grown at permissive temperature. (**D**) Effects of Rtg gene deletions on the survival of *Rho0* versions of *cdc13-1*, Δ*cdc26* and *cdc15-2* cells. (**E**) The survival rates of *cdc13-1*, *cdc13-1* Δ*rtg3* and *cdc13-1 Δras2* double mutants and *cdc13-1 Δrtg3* Δ*ras2* triple mutant. Data are represented as mean +/− SEM.

Is G0/G1 extension the only reason for the cell death-related effects of *Rho0* transformation? The data of Qi et al. [17] point to the opposite. As aforementioned, *Rho+* to *Rho0* conversion in *S. cerevisiae* is known to activate Rtg pathway. To test a possible role of this pathway we deleted *RTG2* or *RTG3* genes in *cdc13-1*, Δ*cdc26* and *cdc15-2* cells and performed the survival test. Surprisingly, the deletions of either gene significantly increased the survival of *cdc13-1*, whereas the effect in the other mutants was either much smaller (*cdc15-2*) or absent (*Δcdc26*) (Figure 4A). Importantly, the doubling times of Rtg-knockout strains are not longer than the ones (3.19 ± 0.14 hours for cdc13-1 vs 2.82 ± 0.12 hours for cdc13-1 Δrtg3) of parental strains. Thus, unlike *Rho0* transformation or deletion of *RAS2*, the deletions of Rtg genes appear neither to increase the G1 duration nor to prevent the entry into S-phase.

It is known that Rtg pathway is negatively regulated by Mks1p transcription factor [22]. Therefore we expected the deletion of *MKS1* to result in the reduction of the survival. To test this, we modified our assay to allow better detection in the decrease of survival: we used YPGly or YPRaf instead of YPD. Under these conditions the survival of *cdc13-1* mutant was above 50%, while the survival of *cdc13-1 Δmks1* double mutant was two to three times lower depending on the media (Figure 4B).

Thus, the deletions of Rtg genes increase the survival and, conversely, the deletion of their suppressor *MKS1* reduces the survival of *cdc13-1* cells. This implies that Rtg pathway is active in the cells under the conditions of the survival test. To test this directly we used the fact that nuclear localization of Rtg3p signals activation of Rtg pathway [33]. To visualize the localization of Rtg3p in this strain, we replaced the native *RTG3* gene with *RTG3*-*GFP*. It appeared that, even at the permissive temperature, the localization of Rtg3-GFP is predominantly nuclear (Figure 4C), indicating that Rtg pathway in *cdc13-1* cells is activated under the standard growth conditions. Most likely this is not due to *cdc13-1* mutation but a common feature of our yeast strain background. Indeed, earlier we found that the *Rho0* mutation did not induce expression of retrograde signaling marker genes in our laboratory strains [34], that can be explained by high basic level activation of Rtg cascade in the used genetic background.

Together, our data suggest that *Rho0* conversion or *RAS2* deletion protect the cells due to the elongation of G0/G1 phase while the deletion of Rtg acts via a different mechanism, the one specific to S-phase. This predicts that inactivation of Rtg pathway would increase the survival of *cdc13-1* or *cdc15-2* cells regardless of the presence of functional mitochondrial DNA or *RAS2*. We tested this prediction, and the results are shown by Figure 4D, 4E. It appeared that the inactivation of Rtg pathway increased the survival only in *cdc13-1 Rho0* double mutants and did not have any effect in *cdc15-2 Rho0* or Δ*cdc26 Rho0*. We observed negative genetic interaction of Δ*rtg3* and Δ*ras2* mutations in *cdc13-1* cells. We explain the latter result by the fact that the deletion of *RAS2* affects many aspects of cell physiology and therefore it was difficult to predict the phenotype of the triple mutant. As for the difference between the effects of *cdc13-1* on one hand and both *cdc15-2* and Δ*cdc26* mutations in the Rtg-minus, *Rho0* backgrounds, most likely they are somehow related to the differences of the pathologies which are caused by the prolong delays in the cell cycle. Indeed, we have detected gross chromosomal rearrangements in *cdc15-2* but not in *cdc13-1* (Figure 2B).

**Figure 5:**
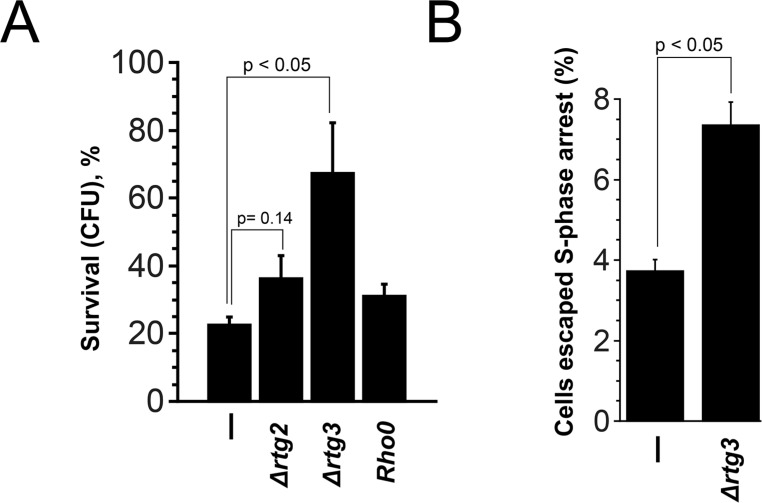
Rtg inactivation but not *Rho0* transformation improves the survival due to alleviation of the strength of the cell cycle arrest (**A**) The survival rates of *cdc13-1*, *cdc13-1 Δrtg2*, *cdc13-1 Δrtg3* and *cdc13 Rho0* cells pre-incubated in HU prior to the 10 h arrest at non-permissive temperature. (**B**) The percentages of *cdc13-1* and *cdc13-1 Δrtg3* which were not in the S-phase after 6 hour incubation at semi-permissive temperature (32°C). The cell cycle stage was determined by microscopy (DIC and DAPI staining), budded cells with two separated nuclei or unbudded cells with single nuclei were counted as non S-phase ones. Data are represented as mean +/− SEM.

To demonstrate directly that the protective effect of Rtg inactivation in *cdc13-1* mutant was not due to the elongation of G0/G1, we synchronized the cells with hydroxyurea at permissive temperature and then performed the survival test. Figure 5A shows that, unlike Δ*ras2* or *Rho0* mutations in *cdc15-2* mutant, the Rtg-minus versions of *cdc13-1* mutant displayed the survival rates higher than the control one. What is the possible protective mechanism induced by inactivation of Rtg pathway? Previously it was shown that the deletions of the DNA-damage checkpoint genes, *RAD24* and several other ones, (which are responsible for the arrest) increase the survival of *cdc13-1* cells at semi-permissive temperature [35, 36]. Therefore we decided to test whether the deletion of *RTG3* gene affects the strength of the arrest. It appeared that, at semi-permissive temperature, the arrest of *cdc13-1* single mutant was significantly stronger than the one of *cdc13-1 Δrtg3* double mutant (Figure 5B). Importantly, at non-permissive temperature *cdc13-1 Δrad24* cells grow into microcolonies [36], pointing that a limited number of cell divisions may not be lethal in the absence of functional Cdc13p.

## DISCUSSION

Our data show that *S. cerevisiae* cells can tolerate long term arrest in G1-phase, but lose the ability for further proliferation if instead arrested in S- or M-phase (see Figure 1). While several works suggest that death in such cases is an active process [16, 17], the available data indicates that the reason for the inability of cells to recover from extremely long non-G1 arrest is mechanical: *cdc13-1*/S-phase arrest -induced death can be prevented by sorbitol [37], and the M-phase arrest is associated with major chromosomal abnormalities (Figure 3). Thus, it appears that the major factor affecting cell survival after excessively long arrest is the ability for checkpoint bypass and continuation of the cell cycle. According to our data mitochondria play a dual role in cell cycle regulation. On one hand, under unfavorable conditions the shortage of mitochondria-supplied energy could prevent the S-phase entry. On the other hand, activated retrograde signaling strengthens the S-phase checkpoints induced by telomere dysfunction.

What are the molecular links between Rtg pathway and DNA damage checkpoint? Possibly, the answer comes from the observation that the deletion of Rtg genes can down-regulate the expression of *SWE1*, a major cell cycle regulating kinase [38]. Swe1p inhibits activity of the main cell cycle-driving kinase, Cdc28p [39]. Consistent with this line of reasoning, Swe1p was shown to accumulate in S-phase arrested yeast cells [40]. Importantly, Swe1p-dependent regulation of cell cycle is conserved in higher organisms. In particular, it was shown that *WEE1*, a mammalian *SWE1* homolog, delays growth after DNA damage thus inhibiting the recovery from cell senescence (see ref. [41] for review).

Our data suggests that activated Rtg signaling (a consequence of dysfunctional mitochondria and/or decreased available ATP levels) in the S-phase activates the checkpoint and delays the cell cycle progression. Importantly, Rtg signaling is involved in maintenance of cellular NTP pools, and the deletions of Rtg genes result in a rapid decrease of nucleotide concentrations [42]. Therefore, under unfavorable conditions, such delay may provide additional time to complete DNA replication.

While mammalian cells can respond to a decrease in ATP levels with their version of retrograde response [43], to our knowledge the question whether the role of Rtg pathway in the checkpoint regulation is conserved in higher eukaryotes is so far not addressed. If something similar exists in higher organisms, than tightening of cell cycle arrest by retrograde signaling under stress conditions can inhibit growth of cells with non-functional mitochondria. As cancer cells typically rely on glycolysis and inhibit mitochondrial energy functions [44], such regulation could be a mechanism that suppresses proliferation of such unwanted cells in multicellular eukaryotes.

## MATERIALS AND METHODS

### Yeast strains and growth conditions

In this study we used strains of *W303* genetic background (Table 1). Yeast cells were grown on rich medium (YPD) according to Sherman et al. [45] at room temperature. Yeast strains used in this study are listed in Table 1.

**Table 1:** Yeast strains used in this study

Strain	Genotype	Source
*cdc13-1*	*MAT a ade2-1 trp1-1 can1-100 leu2-3, 112 his 3-11, 15 ura3 cdc13-1*	[46]
*cdc15-2*	*MAT a ade2-1 trp1-1 can1-100 leu2-3, 112 his 3-11, 15 ura3 cdc15-2*	[47]
Δ*cdc26*	*MAT a ade2-1 trp1-1 can1-100 leu2-3, 112 his 3-11, 15 ura3 cdc26::Kl URA3*	[28]
*cdc34-2*	*MAT a ade2-1 trp1-1 can1-100 leu2-3, 112 his 3-11, 15 ura3 cdc34-2*	[48]
*cdc53-1*	*MAT a ade2-1 trp1-1 can1-100 leu2-3, 112 his 3-11, 15 ura3 cdc53-1*	[48]
*cdc15-2 Δras2*	*MAT a ade2-1 trp1-1 can1-100 leu2-3, 112 his 3-11, 15 ura3 cdc15-2 ras2::TRP1*	This study
*cdc13-1 Δras2*	*MAT a ade2-1 trp1-1 can1-100 leu2-3, 112 his 3-11, 15 ura3 cdc13-1 ras2::TRP1*	This study
*cdc13-1 RTG3-GFP*	*MAT a ade2-1 trp1-1 can1-100 leu2-3, 112 his 3-11, 15 ura3 cdc13-1 TRP1::RTG3-GFP*	This study
*cdc13-1 Rho0*	*MAT a ade2-1 trp1-1 can1-100 leu2-3, 112 his 3-11, 15 ura3 cdc13-1 Rho0*	This study
*cdc15-2 Rho0*	*MAT a ade2-1 trp1-1 can1-100 leu2-3, 112 his 3-11, 15 ura3 cdc15-2 Rho0*	This study
Δ*cdc26 Rho0*	*MAT a ade2-1 trp1-1 can1-100 leu2-3, 112 his 3-11, 15 ura3 cdc26::Kl URA3 Rho0*	This study
*cdc13-1 Δrtg3*	*MAT a ade2-1 trp1-1 can1-100 leu2-3, 112 his 3-11, 15 ura3 cdc13-1 rtg3::KanMX4*	This study
*cdc15-2 Δrtg3*	*MAT a ade2-1 trp1-1 can1-100 leu2-3, 112 his 3-11, 15 ura3 cdc15-2 rtg3::KanMX4*	This study
Δ*cdc26 Δrtg3*	*MAT a ade2-1 trp1-1 can1-100 leu2-3, 112 his 3-11, 15 ura3 cdc26::Kl URA3 rtg3::KanMX4*	This study
*cdc13-1 Δrtg2*	*MAT a ade2-1 trp1-1 can1-100 leu2-3, 112 his 3-11, 15 ura3 cdc13-1 rtg2::KanMX4*	This study
*cdc15-2 Δrtg2*	*MAT a ade2-1 trp1-1 can1-100 leu2-3, 112 his 3-11, 15 ura3 cdc15-2 rtg2::KanMX4*	This study
Δ*cdc26 Δrtg2*	*MAT a ade2-1 trp1-1 can1-100 leu2-3, 112 his 3-11, 15 ura3 cdc26::Kl URA3 rtg2::KanMX4*	This study
*cdc13-1 Δrtg3 Rho0*	*MAT a ade2-1 trp1-1 can1-100 leu2-3, 112 his 3-11, 15 ura3 cdc13-1 rtg3::KanMX4 Rho0*	This study
*cdc15-2 Δrtg3 Rho0*	*MAT a ade2-1 trp1-1 can1-100 leu2-3, 112 his 3-11, 15 ura3 cdc15-2 rtg3::KanMX4 Rho0*	This study
Δ*cdc26 Δrtg3 Rho0*	*MAT a ade2-1 trp1-1 can1-100 leu2-3, 112 his 3-11, 15 ura3 cdc26::Kl URA3 rtg3::KanMX4 Rho0*	This study
*cdc13-1 Δrtg2 Rho0*	*MAT a ade2-1 trp1-1 can1-100 leu2-3, 112 his 3-11, 15 ura3 cdc13-1 rtg2::KanMX4 Rho0*	This study
*cdc15-2 Δrtg2 Rho0*	*MAT a ade2-1 trp1-1 can1-100 leu2-3, 112 his 3-11, 15 ura3 cdc15-2 rtg2::KanMX4 Rho0*	This study
Δ*cdc26 Δrtg2 Rho0*	*MAT a ade2-1 trp1-1 can1-100 leu2-3, 112 his 3-11, 15 ura3 cdc26::Kl URA3 rtg2::KanMX4 Rho0*	This study
*cdc13-1 Δrtg3 Δras2*	*MAT a ade2-1 trp1-1 can1-100 leu2-3, 112 his 3-11, 15 ura3 cdc13-1 rtg3::KanMX4 ras2::TRP1*	This study
*cdc13-1 Δmks1*	*MAT a ade2-1 trp1-1 can1-100 leu2-3, 112 his 3-11, 15 ura3 cdc13-1 mks1::KanMX4*	This study
*cdc15 CENV_GFP*	*MAT a leu2::tetR-GFP-LEU2 HIS3-112 tetO2 (integrated 1.4kb left on CENV) ade2-1 trp1-1 can1-100 his3-11, 15 ura3 cdc15-1*	This study
*cdc13 CENV_GFP*	*MAT a leu2::tetR-GFP-LEU2 HIS3-112 tetO2 (integrated 1.4kb left on CENV) ade2-1 trp1-1 can1-100 his3-11, 15 ura3 cdc13-1*	This study
*W303 CENV_GFP*	*MAT alpha leu2::tetR-GFP-LEU2 HIS3-112 tetO2 (integrated 1.4kb left on CENV) ade2-1 trp1-1 can1-100 his3-11, 15 ura3 cdc13-1*	[31]

### Genetic screening

Transposon-mutagenized yeast cells (according to Burns et al. [49]) of *cdc15*-*2* strain were transferred to selective synthetic media plates (10^6^ cells per plate). The plates were incubated at 37°C for 24 hours, then at room temperature for 72 hours. Viability of the cells according to CFU was ∼0.1%. Grown colonies were collected (washed out with fresh medium) and subjected to one more round of screening. After that we selected individual colonies and tested whether viability of these clones during prolonged incubation at restrictive temperature was higher than the one of the parental strain. Next, we mapped transposon insertion position in selected clones to determine the genes which were disrupted by the insertions. For further experiments we independently deleted the genes of interest in the parental strains.

### Survival experiments

Survival of yeast cells arrested in the different stages of the cell cycle was measured by CFU method. For this purpose yeast cells were grown overnight on fresh YPD plates and then transferred to a fresh YPD plate in a set of dilutions. In case of hydroxyurea presynchronization, yeast cells were grown overnight in liquid YPD, then hydroxyurea was added to the medium to a final concentration of 150 mM and the cells were incubated at 28°C for 5 hours. Then the plate was thoroughly sealed with Parafilm to avoid drying (evaporation of water from media significantly altered the results of the experiments). After this yeast cells were incubated at restrictive temperature (37°C) for indicated periods of time and after that at room temperature for 48 hours.

### Microscopy

Fluorescence microscopy was used (1) to determine GFP-fusion protein localization, (2) to count the number of centromeres (3) to measure viability with propidium iodide and (4) to count the cells that escaped S-phase arrest. Yeast cells were visualized using an Olympus BX51 microscope (U-MNIBA3 filter for GFP, U-MNU2 filter for DAPI and U-MNG2 filter for propidium iodide). Photographs were taken with a DP30BW CCD camera.

### Statistical analysis

All data are presented as averages and standard errors. Wilcoxon rank-sum unpaired test was used to compare datasets from different strains or conditions with the R software package. *indicates *p*-value < 0.05, **p-value < 0.01.
